# Comparison of *in vitro* digestibility and chemical composition among four crop straws treated by *Pleurotus ostreatus*

**DOI:** 10.5713/ajas.18.0023

**Published:** 2018-05-31

**Authors:** Haitao Nie, Ziyu Wang, Jihao You, Gang Zhu, Hengchang Wang, Feng Wang

**Affiliations:** 1 Deparment of Jiangsu Engineering Technology Research Center of Meat Sheep & Goat Industry, Nanjing Agricultural University, Nanjing 210095, China; 2 Animal Husbandry and Veterinary Station of Guannan, Lianyungang 222500, China

**Keywords:** Goats, *In vitro* Digestibility, Nutrient Availability, Pleurotus ostreatus, Crop Residues, Biodegradation

## Abstract

**Objective:**

The effects of *Pleurotus ostreatus* on the feed utilization of broad bean stalks (BBS), rape straw (RS), paddy straw (PS), and corn stalk (CS) was examined.

**Methods:**

The four roughages were co-cultured with *Pleurotus ostreatus*. The chemical composition; enzyme activities of laccase, carboxymethylcellulase (CMCase) and xylanase; carbohydrate and protein fractions (based on The Cornell Net Carbohydrate and Protein System [CNCPS]) were assessed at different days after inoculation (7, 14, 21, 28 d) and un-inoculated roughages (control, 0 d). The digestibility of nutrient components and the gas production of roughage with various incubation times were monitored at 0, 2, 4, 6, 9, 12, 24, 36, 48, 60, and 72 h using an *in vitro* ruminal fermentation method.

**Results:**

A higher CMCase activity (0.1039 U/mL) and earlier time to peak (14 d) were detected in *Pleurotus ostreatus* cultured with CS (p<0.05). Significantly, the incubation length-dependent responses of cumulative gas production were observed from 24 to 72 hours post fermentation (p<0.05), and these incubation length-dependent effects on cumulative gas production of PS and CS appeared earlier (24 h) for PS and CS than those (48 h) for BBS and RS (p<0.05). The fast-degradable carbohydrate (CA) content for all four roughages significantly increased over time (p<0.05). Nonetheless, increased degradation efficiency for CA treated with *Pleurotus ostreatus* was detected at both 21 and 28 days of incubation (p<0.05). With the exception of PS (p<0.05), there were no significant difference among the roughages (p>0.05) in slowly-degradable carbohydrate (CB2) at different incubation times (p<0.05).

**Conclusion:**

Assessment of the alterations in chemical composition, CNCPS system fractions, and the fermentation kinetics after biological pretreatment may yield a valuable database for evaluating the biological pretreatment of *Pleurotus ostreatus* in ruminant feed.

## INTRODUCTION

Most research on ruminant feed has focused on finding alternative feed ingredients (e.g. straws and other crop residues) that can replace conventional ones (e.g. grasses, grains, and beans) [1]. Rape seed, broad bean, rice, and maize are widely planted all over the world. The feed utilization of their straws as feed is of great importance in the herbivore livestock husbandry, which can both alleviate forage shortage and reduce straw incineration [2]. Although crop straws are abundant and low-cost roughage resource for feed ruminants, these animals can only partially utilize straw fibers (cellulose and hemicellulose, but not lignin) [3]. The high lignin content is a major constraint for the use of crop straw as a feedstuff, because lignin forms insoluble polymers with cellulose and hemicellulose or it can form an insoluble matrix [4]. Moreover, lignin reduces the digestibility of the crop straw through special interconnections with other polymers, since these bonds that are not hydrolysable under normal biological conditions [5]. Consequently, improving the digestibility of crop straws has become an important goal in ruminant nutrition research. Numerous physical and chemical methods have been explored in order to degrade the lignocellulosic bonds and increase the nutrients availability of crop straw, however the bio-conversion methods appear to be the most promising [6].

White-rot fungi are able to decompose all wood fractions, including lignin, and leave the wood with a white fibrous appearance [7]. This process occurs as a result of the ability of these fungi to produce a variety of enzymes that are involved in lignin degradation and the destruction of physically tough plant material. The Biological pretreatment of lignocellulosic biomass by white-rot fungi can represent a low-cost and eco-friendly alternative physicochemical methods to facilitate enzymatic hydrolysis. *Pleurotus ostreatus* is a white rot fungi that is well known as being an edible and delicious mushroom. It can be cultivated on both lignin and cellulose-containing substrates since it has lignin lytic system [8].

The Cornell Net Carbohydrate and Protein System (CNCPS) is a dynamic model of fermentation in nutrition, and combined with a chemical analysis of feed with cell ingredients of plant and digestibility of the ruminant [9]. This system has been used to estimate the animal requirements and nutrient supply under different breeding, environmental condition, and feed composition. For ruminants, CNCPS contains a sub-model for predicting the rates of feedstuff degradation in the rumen, and the transport of un-degraded feed to the lower gut, and the amount of variable protein and energy. This model was developed based on the acid and neutral detergent system of feed analysis for both for carbohydrate and protein fractions. Based on the analyses of chemical fractions and fermentative characteristics of the rumen, the feed carbohydrate is divided into CA (fast fermented), CB1 (moderate fermented), and CB2 (slowly fermented), and CC (unfermentable). In addition, the CNCPS assigns a fixed rate of degradation (%/h) to each protein fraction, which is independent of the feed in which feed they occur in, namely infinity, 200% to 300%, 5% to 15%, 0.10% to 0.15%, and 0 for fractions PA (non-protein nitrogen), PB1 (rapidly degradable protein), PB2 (intermediately degradable protein), PB3 (slowly degradable protein), and PC (unavailable protein) respectively. Taken together the application of CNCPS reflects not only the carbohydrate composition but also the potential fermentative activity of the rumen.

Previous studies have focused on the degradative action of white rot fungi [10], but few researches evaluate the enhancement of digestibility and nutrition loss from the perspective of ruminal degradation, which may be key points in crop straws utilization. In the present study, we determined the enzyme activities and changing patterns of *Pleurotus ostreatus* following co-culture with different roughage types or culturing times. For all samples, chemical composition and the CNCPS fraction digestibility, and gas production were evaluated using an *in vitro* ruminal fermentation method in order to provide a valuable database for the biological pretreatment of crop straws with white-rot fungus for ruminant feed.

## MATERIALS AND METHODS

### Animal care

All experiments were conducted in accordance with ethical procedures and policies approved by Nanjing Agricultural University Animal Care Committee (SYXK2011-0036).

### Preparation of the substrates and fungal strains inoculation

The *Pleurotus ostreatus* strain was obtained from the Animal Science Institute of Jiangsu Academy of Agricultural Sciences (Nanjing, JiangSu, China). The stock cultures were maintained on potato dextrose agar (PDA) in test tube slants at 4°C. The substrate samples of broad bean stalks (BBS), rape straw (RS), paddy straw (PS; round-grained nonglutinous rice) and corn stalk (CS) were collected from HaiMen (NanTong, JiangSu, China; Northern latitude 31°53′, Eastern longitude 120°09′; harvested on May 21), TaiZhou Country (JiangSu, China; Northern latitude 32°26′, Eastern longitude 120°14′; harvested on May 4), ChongMing Country (NanTong, JiangSu, China; Northern latitude 31°47′, Eastern longitude 121°25′; harvested on October 23), and SuiNing Country (XuZhou, JiangSu, China; Northern latitude 33°54′, Eastern longitude 117°56′; harvested on October 6) respectively. The roots of the collected samples were removed, and the residue samples were then oven dried at 65°C for 24 h. Thereafter, the four types of roughage were mechanically milled and sieved through a 40-mesh screen to provide sufficient contact between the fungal strain and substrate. For each treatment replicate, 20 g of the mashed stalk and 50 mL of distilled water, which were sterilized by autoclaving in a 250-mL Erlenmeyer flask for 30 min at 120°C in autoclave, cooled, and then inoculated at 28°C±2°C with 75 mL of PDA that was inoculated by three mycelial discs (8 mm). Samples were taken for analysis and further investigation at different times of incubation (7, 14, 21, and 28 d), in addition to the un-inoculated roughages (control, 0 d).

### Rumen liquor collection

At 1.5 h after feeding, rumen liquor samples were obtained from mature Yangtze River Delta white male goats (n = 4; body weight = 42.36±1.27) fed a total mixed ration comprising 94 g/kg dry matter (DM) crude protein (CP) and 10.27 MJ/kg DM based on the recommendation for growing goats [11]. Following this, the contents were squeezed through eight layers of cheese cloth and purged with O2-free carbon dioxide. Finally, large feed particles and protozoa were removed by slow-speed centrifugation (150×g for 5 min at 15°C).

### Assay of activities of laccase, carboxymethylcellulase, and xylanase enzymes

Laccase activity was determined by measuring the change in oxidation of 2,2′-azinobis-3-ethylbenzo-thiazoline-6-sulfonate (ABTS) [12]. The procedure was conducted in 3 mL reaction mixtures consisting of 2.7 mL of 0.1 mol/L sodium acetate buffer, 0.2 mL of 1 mmol/L ABTS solution, and 0.1 mL of extracellular culture supernatant. The reaction was monitored by measuring the change in absorbance at 420 nm for 3 min. Carboxymethylcellulase (CMCase) activity was determined using 1% carboxymethyl cellulose (CMC) as the sole carbon source. The reaction mixture was comprised of 0.2 mL of enzyme sample and 1.8 mL of CMC (1%) dissolved in 0.1 mol/L of sodium acetate buffer (pH 4.8). The reaction was performed at 50°C for 30 min. Xylanase activity was determined by measuring the release of reducing sugars, as described by Meddebmouelhi et al [13]. The enzyme activities of laccase, CMCase, and xylanase, were assessed at different inoculation times (7, 14, 21, and 28 d) as well as for un-inoculated roughages (control, 0 d). The values of enzyme activity were expressed as U/mL and defined as the amount capable of producing one μg of product per minute per mL of extracted substrate.

### *In vitro* ruminal fermentation

The *in vitro* ruminal digestibility of organic matter, neutral detergent fiber, and gas production were determined according to the method described by Tilly [14] with slight modifications. Samples were placed in an incubator with a capability of holding 204 bottles (Forma Scientific, model 39419-1, Marietta, OH, USA), at 39°C±0.5°C. The bottles were shaken using a rotary shaker platform at 120 oscillations/min (Lab-Line Instruments Inc, Melrose Park, IL, USA). On the day before incubation, 0.5 g DM of each forage sample (n = 2) was weighed into each bottle (n = 3 for each treatment). On the day of incubation, the fermentation bottles were fitted with a side port, and sealed with a screw cap fitted with a gas-tight septum, as previously demonstrated [15]. The bottles were prepared under an atmosphere of CO_2_ to ensure anaerobic conditions, a buffered rumen liquor solution was added (20 mL) to each bottle that was then incubated in shaking water bath (Hake SWB25, Clausthal-Zellerfeld, Germany) at 39°C for 72 h and connected to an automated gas production system. Gas production was measured after 0, 2, 4, 6, 9, 12, 24, 36, 48, 60, and 72 h of incubation. For each roughage substrate, 33 bottles (3 replicates×10 collection points plus 3 controls) were removed from the incubator, and the gas production from each bottle was immediately collected using a water displacement apparatus.

The fermentation kinetics were determined based on the gas production curve, and fitted to the following model according to Schofield et al [16] as follows: Vt = *Vf*×(1−exp [−*K*× {t−*Ln*}]); where the Vt represents the volume of gas produced (mL) up to time t (h); *Vf* represents the maximum gas produced (mL) after the asymptote is reached; *K* is the fractional rate of gas production (1/h); and *Ln* represents the discrete lag phase (h).

### Chemical composition, protein and carbohydrate fraction calculation based on CNCPS

The CP, crude fat, total ash non-protein nitrogen (NPN), and starch were determined following the AOAC [17]. Cellulose, hemicellulose, lignin, acid detergent-insoluble CP (ADICP), and neutral detergent insoluble CP (NDICP) were determined according to the method described by Van Soest [18]. Soluble protein (SOLP) was measured based on the method described by Krishnamoorthy [19].

The original CNCPS protein fractionation method divides the feed protein into five fractions: i) NPN [PA(% DM) = NPN×0.0001×SOLP×CP], which is the N soluble in buffer and not precipitated by protein precipitating agents such as trichloroacetic acid, and contains peptides, free amino acide, ammonia, amides, amines, ureides, nucleotides, and nitrates; ii) rapidly degradable protein [PB1 (% DM) = SOLP×CP×0.01–PA)], which is assumed to be very rapidly degraded in the rumen with degradation rates greater than 1.0/h; iii) intermediately degradable protein ([PB2 {% DM} = CP–PA–PB1–PB3–ADICP]), which represents the intermediate degradable protein with rates of degradation within the range 0.03 to 0.16/h; iv) slowly degradable protein [PB3 (% DM) = (NDICP− ADICP)×CP×0.01], which describes the CP insoluble in neutral detergent solution, but soluble in acid detergent; v) unavailable protein [PC (% DM) = ADICP×CP×0.01], which is assumed to be the protein associated with lignin, tannin-protein complexes, and MAILLARD reaction products. Additionally, the fast-degradable carbohydrate (CA [% DM] = DM–neutral detergent fiber [NDF]–neutral non-soluble protein [NDFIP]–2×LIGNIN×2.4–STARCH), intermediate degradable carbohydrate [CB1 (% DM) = STARCH], slowly degradable carbohydrate [CB2 (% DM) = NDF–NDFIP–LIGNIN×2.4], and unavailable degradable carbohydrate [CC (DM) = LIGNIN×2.4] were determined according to the classification of carbohydrates fractions based on degradation rate described by CNCPS.

### Statistical analyses

Statistical analyses were performed using the SPSS (19.0). The data are presented as mean and standard error of the mean (SEM), and the differences are regarded as significant at p< 0.05. Two-way analysis of variance (ANOVA) with roughage type as one variable and incubation time as the other variable was carried out to study the fixed effects of roughage substrate types and incubation time, for the data not only involved in chemical composition, CNCPS fractions content and *in vitro* fermentation kinetics of roughage at different incubation times, but also consisted of the data of enzyme activities of *Pleurotus ostreatus* co-cultured with different roughage types. For the data within the same roughage type or incubation time, data were analyzed for treatment effect using one-way ANOVA. Post-hoc differences between different groups were further examined using Tukey’s test.

## RESULTS

### The activities of laccase, carboxymethylcellulase, and xylanase enzymes

**Figure 1:**
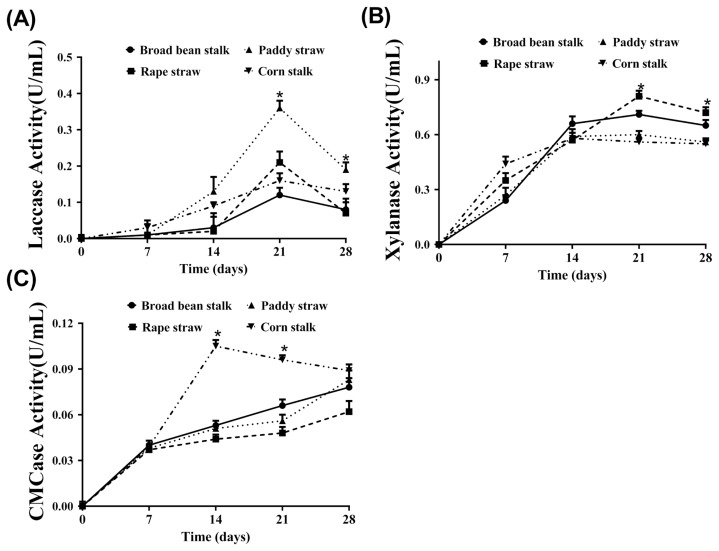
Crucial degrading enzymes activities of *Pleurotus ostreatus* cultured with Broad bean stalk (● and ▬), Paddy straw (▲ and ···), Rape straw (■ and ---), and Corn straw (▼ and –··–) at 0, 7, 14, 21, and 28 day of co-culturing time. (A) Enzyme activity of laccase of *Pleurotus ostreatus*; (B) Enzyme activity of Xylanase of *Pleurotus ostreatus*; (C) Enzyme activity of carboxymethylcellulase of *Pleurotus ostreatus*. The symbolic of * indicates significant different (p<0.05).

The enzyme activities of *Pleurotus ostreatus* (laccase, CMCase, and xylanase) co-cultured with RS, BBS, PS, and CS for different cultivation times (0, 7, 14, 21, and 28 d of incubation) are represented in Figure 1. The laccase activity of *Pleurotus ostreatus* incubated with PS was significantly greater (0.3658 U/mL at 21 d; p<0.05) than those that were co-cultured with the other types of straws after different times of incubation (Figure 1A). As illustrated in Figure 1B, the xylanase activities of *Pleurotus ostreatus* cultured with BBS, CS, and PS increased significantly from 0 to 14 d (p<0.05) and were then maintained at a relatively stable level from 14 to 28 d of incubation. Nevertheless, the xylanase activity of treated RS increased remarkably during the first 21 days (p<0.05) and were then maintained at a relatively stable level from 21 to 28 d. In addition, compared with *Pleurotus ostreatus* co-cultured with BBS, RS, and PS, a higher CMCase activity (0.1039 U/mL) and an earlier time to peak time (0.1039 U/mL at 14 d; p<0.05) were detected in *Pleurotus ostreatus* co-cultured with CS (Figure 1C).

### *In vitro* ruminal fermentation

**Figure 2:**
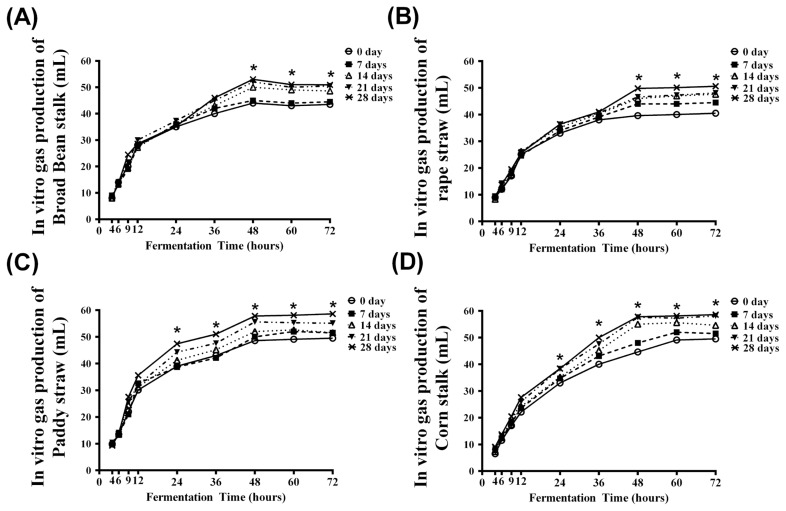
*In vitro* ruminal fermentation of stalks cultured with *Pleurotus ostreatus* for 0, 7, 14, 21, and 28 day of co-culturing time. (A) *In vitro* gas production of broad bean stalk degraded by *Pleurotus ostreatus* for different time; (B) *In vitro* gas production of rape straw degraded by *Pleurotus ostreatus* for different time; (C) *In vitro* gas production of paddy straw degraded by *Pleurotus ostreatus* for different time; (D) *In vitro* gas production of corn stalk degraded by *Pleurotus ostreatus* for different time. The symbolic of * indicates significant different (p<0.05).

The cumulative gas productions of RS, BBS, PS, and CS incubated with *Pleurotus ostreatus* for various days (0 to 28 d of incubation periods (0 to 28 d) relative to the different *in vitro* ruminal fermentation times (0 to 72 h of fermentation) are illustrated in Figure 2. For each roughage, the cumulative gas production at 4, 6, and 9 h of fermentation were not significantly affected by the incubation times. Significant responses to the time of incubation (p<0.05) were observed at 24 h for PS (Figure 2C) and CS (Figure 2D), compared to BBS (Figure 2A) and RS (Figure 2B), for which effects did not appear until 48 h post fermentation.

The fermentation kinetics of RS, BBS, PS, and CS treated with *Pleurotus ostreatus* for different days are presented in Table 1. The maximum substrate digestion (*Vf*) for all roughage types increased with incubation time (p<0.05). The significant effects of roughage type on *Vf* were detected from 14 to 28 d of incubation (p<0.05), over this incubated period, the *Vf* values of CS at 14, 21, and 28 d of incubation were all significantly greater than that of RS (p<0.05). The *Ln* values of BBS and CS were decreased significantly with increasing time (p<0.05), whereas in contrast, the *Ln* values of RS and PS increased significantly with increasing time (p<0.05).

### The chemical composition of roughages treated with *Pleurotus ostreatus*

The chemical composition of RS, BBS, PS, and CS co-cultured with *Pleurotus ostreatus* for different incubation days are presented in Table 2. Independent of the roughage type, the CP content significantly increased with incubation time (p<0.05). Nonetheless, the carbohydrates (CHO) were not significantly affected by substrate type or incubation time. With the exception of RS, the cellulose content decreased significantly with incubation time (p<0.05). A significantly increased hemicellulose content was detected in PS and CS only with incubation time (p<0.05). However, the hemicellulose contents of BBS and RS were similar across the entire incubation period. The lignin content of four substrates all significantly decreased with incubation time (p<0.05).

### CNCPS protein fractions of roughages treated with *Pleurotus ostreatus*

The CNCPS protein fractions of RS, BBS, PS, and CS that co-cultured with *Pleurotus ostreatus* for different incubation times are presented in Table 3. The PA and PB1 fractions of BBS significantly decreased with increasing time (p<0.05). Whereas there was no significant effect of incubation days on PA or PB1 fractions of RS, PS, and CS. There was a significant effect of both incubation time and roughage type on PB2 fractions, which increased significantly with incubation time (p<0.05) independent of the type of substrates. Additionally, the PB2 contents of BBS and RS were significantly greater (p<0.05) than those of PS and CS (p<0.05) independent of the incubation days. The PB3 contents of the four substrates were not affected by the type of roughage or by incubation time. With the exception of PS, the PC contents of all the other roughages significantly decreased with increasing incubation time (p> 0.05). Additionally, the PC fraction of CS decreased significantly in day 28 of incubation, and the PC fraction of CS was lowest than those of other roughages at all incubation days (p<0.05).

### CNCPS carbohydrate fractions of roughages treated by *Pleurotus ostreatus*

The CNCPS carbohydrate fractions of RS, BBS, PS, and CS that co-cultured with *Pleurotus ostreatus* for different incubation times are presented in Table 4. The CA contents of the four substrates were all significantly increased over the time of incubation (p<0.05), whereas the relatively improved degradation efficiency for CA was detected in PS treated PS after 21 to 28 d of incubation (p<0.05). Significant effects of incubation time on CB1 content were detected in both BBS and PS (p<0.05). In contrast, the CB1 contents of RS and CS were similar over time. With the exception of PS (p<0.05), there were no significant differences among the different roughages in CB2 over time. Independent of the type of roughage type, the CC of all the roughages all decreased significantly over time (p<0.05).

## DISCUSSION

The lignocellulosic biomass in crop stalks is produced in large quantities, which represents a great potential as a feedstock. Generally, lignin hinders the digestion of lignocellulosic biomass by ruminants. The lignin cannot be easily degraded by a lignin degrading enzyme only, but requires the synergistic action of several enzymes, including cellulose and hemicellulose enzymes [20]. As a white rot fungus, *Pleurotus ostreatus* has an active ligninlytic system [8], and can produced variable enzyme activities depending on the circumstances related to the complexity of the roughage itself [21]. The nitrogen concentration in the culture medium of white rot fungi, either in solid-state and submerged fermentation, plays an important role in the production and activity of liginolytic enzymes [22]. An earlier study examining the effect of an inorganic nitrogen source (INS) on the activities of ligninolytic enzymes (laccase, MnP and peroxidase) activities in *Pleurotus ostreatus*, suggested that the high and low concentrations of INS have negative and positive effects on these enzymes respectively [23]. In the present research, the laccase activities of treated RS at 21 and 28 d were significantly greater than that detected for other roughage, we suggested that the relatively lower CP concentration in RS might attributed to the greater production and activity of liginolytic enzymes.

Hemicellulose is more easily degraded compared to other components in the lignocellulosic biomass [7]. It has been reported that the hemicellulose biodegradable rates of crop stalks treated with different species of white-rot fungi ranged from 24.4% to 34.9% [24]. In our study, only the hemicellulose degradation of PS (26.33%) was close to this, being significantly higher than those of BBS, RS, and CS (6.38%, 5.07%, and 9.96%; respectively). This divergence in hemicellulose degradation might be related to the different cellulose polymers in the cell walls, which arise a result of the variety of physical and morphological structures among different types of roughage. In the same context, our data indicate that the cellulose of RS was barely degraded (from 35.94% to 33.86%), the corresponding degradation rate (approximately 8.57%) was less than the other roughages. It has been acknowledged that the lignin/cellulose loss ratio is regarded as a selective value to evaluate the selective lignin-degrading ability [25]. Based on this, the relatively greater value of lignin/cellulose loss ratio for RS (1.23%) suggested a higher selective lignin-degrading ability, which did not affect the RS than that on the other roughage. Therefore, we further suggested that a greater selective lignin degradation of *Pleurotus ostreatus* treated RS compared with other roughages, without removing large amounts of cellulose.

It is well-known that the suitability of the *in vitro* gas test technique for gas production measurements depends on the CP amount in the feedstuffs examined. This is because nitrogenous compounds related to acid-base reaction and rumen pH, indirectly influence gas production [26]. In the present study, the CP content of the four roughages with various incubation times were within normal ranges (4.76% to 10.10%). The gas production curves were typical in shape, and included a lag phase at the beginning, an exponential phase, followed by a plateau phase. The *in vitro* gas production profiles from the different roughages were identical at the beginning of the fermentation. In ruminants, rumen bacteria, fungi, and protozoa can mainly decompose the feed cellulosic substances [27]. The extent of this anaerobic fermentation process depends on the amount of lignin in the feed. In additional, an evaluation of the dynamic parameters of gas production showed that the *Ln* value (discrete lag phase) for BBS and CS significantly decreased with incubation times, indicating that BBS and CS could be more easily utilized than RS and PS, which both had a significantly increased *Ln* with incubation times. This phenomenon might be attributed to the incomplete degradation of lignin, and the endogenous consumption of nonstructural carbohydrates by *Pleurotus ostreatus*. Theoretically, maximum gas production has a negative correlation with lignin content in roughage. Therefore, in the present study, the *Vf* values for all types of roughage all increased with increasing incubation times, which might be related to the decrease in lignin content as incubation time increased. Collectively, this aspect of the research suggests that the roughages treated with *Pleurotus ostreatus* could be better utilized by animals owing to their direct effect on the feed lignin complex.

The NPN plays an important role in rumen digestion since microorganisms can convert ammonia nitrogen, peptides, amino acids to bacterial proteins, and essential amino acid. The results of this study demonstrated that the highest amount of NPN is detected in CS, indicating a greater potential resource pool for bacterial protein and essential amino acids than the other roughages. Additionally, it was acknowledged that if the feedstuff substrate is of high quality, as much as two-thirds of the NPN should be included in the PB2 [28]. In the present study, BBS was the only one substrate that was significantly decreased in PA and increased and PB2 respectively with incubation progress. This might be related to protein interconversion between PA and PB2 as a result of *Pleurotus ostreatus* activity. In addition, for most feeds, the PB2 fraction has been shown to represent the largest protein pool size [9], which is similar to our data showing that the amount of PB2 in the four feedstuffs was greater than those of other CNCPS protein fractions. In addition, PB3 was the only protein fraction that was not affected by incubation time in the present study, and this result is in agreement with the conclusion that degradation rates of the PB3 fraction are virtually zero due and is predicted to almost completely escape from digestion in the rumen [29]. PB3 is associated with the plant cell wall [30], which contains glycoprotein as one of the constituents attached covalently to cellulose fibrils. Although lignin was found to be degraded partially degradable in this study, the amount of PC was not affected. One possible explanation for this was that the lignin matrix was not as well decomposed as PC. This suggestion is in accordance with Ghoorchi and Arbabi [29], who found that the proportional concentrations of NPN and true protein was one of the most important aspects of feed CP utilization.

The CHO are the largest component of rations, and can be partitioned into fiber carbohydrates (FC) and non-fiber carbohydrates (NFC) [27], which vary widely between different feedstuffs. Additionally, the content of FC and NFC are also differ in the rate of fermentation, extent of fermentation, products of fermentation, and the contribution to microbial CP production [31], as lignin is a heterogeneous polymer that occurs in woody and vascular tissues [32]. The colonization with white rot fungi is considered to be a promising technique because of its preferential degradation of lignin. Some degraded parts of CC (mainly lignin) can be converted into carbon dioxide and water, and it is possible that other parts could be transformed into CA or CB1, which is consistent with our results of a decrease in CC and increase in CA for all substrates, and an elevated CB1 content specifically in BBS and PS. The FC (i.e., hemicelluloses and celluloses) is the slowly digestible fraction of feeds that occupies space in the gastrointestinal tract. FC is associated with lignin resistant digestion, and therefore does not contribute significantly to energy in animals. Hemicellulose, one of the major components of the cell wall, had a similar trend to the four roughages with respect to CB2. However, given the absence of a significant difference, which was inconsistent with a high laccase and xylanase enzyme activity in BBS and RS, this divergence might be attribute that the enzyme of laccase and xylanase does not degrade the cell wall exclusively.

## CONCLUSION

Overall, the degradation efficiency improvement by P*leurotus ostreatus* on the *in vitro* digestibility of roughage, using RS, BBS, PS, or CS was demonstrated in this study. Changes in enzymes activities changing patterns of *Pleurotus ostreatus* when co-cultured with different roughage types or culturing times, chemical composition, CNCPS fractions and *in vitro* fermentation kinetics of roughage, demonstrated that *Pleurotus ostreatus* have a good capability to improve the digestibility of roughage resources. This study might promise a microbiological treatment that can improve the nutritive value and ruminal digestibility of poor quality roughage. An assessment of alteration in chemical composition, CNCPS system fractions and the fermentation kinetics after biological pretreatment may yield a valuable database for the application of *Pleurotus ostreatus* in animal feed.

## Figures and Tables

**Table 1:** Dynamic parameters of gas production for various types of roughage cultured with *Pleurotus ostreatus* for different incubation times

Items1)	Substrates	Incubation times (d)					SEM	p-value

		0	7	14	21	28		
*Vf* (mL/g DM)	BBS	47.55^c^	47.49^c^	53.40^AB,a^	55.12^B,a^	55.74^BC,a^	0.97	<0.05
	RS	43.14^c^	47.63^b^	48.10^B,ab^	48.44^C,ab^	51.39^C,a^	0.92	<0.05
	PS	50.08^c^	52.38^bc^	55.94^AB,b^	57.04^B,ab^	59.98^AB,a^	0.91	<0.05
	CS	49.54^c^	52.61^c^	58.69^A,ab^	62.92^A,a^	63.04^A,a^	1.19	<0.05
	SEM	2.67	1.95	2.29	2.34	1.56	-	-
	p-value	<0.05	<0.05	<0.05	<0.05	<0.05	-	-
*K* (h)	BBS	0.059^BC,ab^	0.061^AB,a^	0.055^AB,bc^	0.053^AB,c^	0.054^AB,bc^	0.002	<0.05
	RS	0.061^A,a^	0.055^AB,bc^	0.054^AB,bc^	0.056^AB,b^	0.053^AB,c^	0.001	<0.05
	PS	0.069^A,a^	0.066^A,b^	0.063^A,c^	0.063^A,c^	0.065^A,bc^	0.0008	<0.05
	CS	0.050^C,a^	0.049^B,a^	0.044^B,b^	0.043^B,b^	0.045^B,b^	0.001	<0.05
	SEM	0.003	0.004	0.006	0.004	0.003	-	-
	p-value	<0.05	<0.05	<0.05	<0.05	<0.05	-	-
*Ln* (h)	BBS	0.140^B,c^	0.157^AB,b^	0.179^B,a^	0.103^B,d^	0.105^B,d^	0.005	<0.05
	RS	0.105^B,b^	0.104^B,b^	0.126^B,a^	0.124^B,a^	0.114^B,ab^	0.005	<0.05
	PS	0.278^A,bc^	0.261^A,c^	0.321^A,b^	0.318^A,b^	0.452^A,a^	0.014	<0.05
	CS	0.379^A,a^	0.210^AB,b^	0.138^B,c^	0.154^B,c^	0.153^B,c^	0.014	<0.05
	SEM	0.039	0.034	0.028	0.041	0.026	-	-
	p-value	<0.05	<0.05	<0.05	<0.05	<0.05	-	-

SEM, standard error of the mean; BBS, broad bean stalk; RS, rape straw; PS, paddy straw; CS, corn stalk.

1) The *Vf* represented maximum gas production (mL) after the asymptote is reached; the *K* is fractional rate of gas production (1/h); *Ln* represented discrete lag phase (h).

Significant differences are indicated with capital letters in the same column and small letters in the same row; different letters mean significant difference (p<0.05).

**Table 2:** The chemical composition (DM base) of broad bean stalks, rape straw, paddy straw and corn stalk cultured with *Pleurotus ostreatus* for different incubation times

Items	Substrates	Incubation times (d)					SEM	p-value

		0	7	14	21	28		
CP (%)	BBS	7.35^A,c^	8.09^A,b^	8.15^A,b^	9.03^A,a^	9.31^A,a^	0.19	<0.05
	RS	4.76^B,c^	5.27^B,b^	5.31^B,ab^	5.41^B,ab^	5.55^B,a^	0.08	<0.05
	PS	5.49^B,b^	5.89^B,a^	5.78^B,a^	5.97^B,a^	5.97^B,a^	0.06	<0.05
	CS	7.97^A,d^	8.54^A,cd^	9.06^A,bc^	9.47^A,b^	10.10^A,a^	0.21	<0.05
	SEM	0.49	0.56	0.44	0.42	0.38		
	p-value	<0.05	<0.05	<0.05	<0.05	<0.05		
CHO (%)	BBS	86.87	85.51	85.82	84.83	84.47	0.46	NS
	RS	84.14	83.03	83.69	82.59	82.85	0.47	NS
	PS	85.91	84.81	84.02	85.03	85.53	0.43	NS
	CS	83.83	83.46	83.44	82.33	82.89	0.44	NS
	SEM	1.68	1.77	1.62	1.71	1.63	-	-
	p-value	NS	NS	NS	NS	NS	-	-
Cellulose (%)	BBS	37.02^A,a^	36.41^A,a^	33.31^A,b^	29.36^A,c^	29.50^A,c^	0.94	<0.05
	RS	35.94^A,a^	33.64^B,ab^	33.52^A,ab^	33.55^A,ab^	33.86^A,b^	0.42	NS
	PS	34.48^AB,a^	33.95^B,a^	31.86^A,ab^	31.44^A,ab^	27.41^A,b^	0.84	<0.05
	CS	30.20^B,a^	28.51^B,a^	26.63^B,a^	26.44^B,a^	22.49^B,c^	0.8	<0.05
	SEM	1.06	0.43	0.84	0.98	0.77	-	-
	p-value	<0.05	<0.05	<0.05	<0.05	<0.05	-	-
Hemicellulose (%)	BBS	18.19^C^	17.82^C^	17.79^B^	17.21^C^	17.03^B^	0.2	NS
	RS	23.86^C^	22.87^C^	23.07^B^	22.12^B^	22.65^B^	0.31	NS
	PS	34.67^A,a^	29.36^A,b^	26.07^A,bc^	26.55^A,bc^	25.54^A,c^	0.98	<0.05
	CS	27.11^B,a^	26.90^B,ab^	26.36^A,abc^	24.94^AB,bc^	24.41^A,c^	0.37	<0.05
	SEM	0.21	0.34	0.8	0.37	0.43	-	-
	p-value	<0.05	<0.05	<0.05	<0.05	<0.05	-	-
Lignin (%)	BBS	15.69^A,a^	14.5^A,ab^	12.44^A,bc^	11.27^A,c^	9.94^B,d^	0.8	<0.05
	RS	14.87^A,a^	12.07^A,b^	12.52^A,ab^	12.70^A,ab^	11.06^A,b^	0.43	<0.05
	PS	17.71^A,a^	17.00^A,a^	15.74^A,a^	14.97^A,ab^	11.12^A,b^	0.77	<0.05
	CS	11.88^B,a^	10.94^A,ab^	9.39^A,bc^	8.89^B,c^	8.08^B,c^	0.43	<0.05
	SEM	0.71	0.74	0.8	0.67	0.63	-	-
	p-value	<0.05	<0.05	<0.05	<0.05	<0.05	-	-

DM, dry matter; SEM, standard error of the mean; CP, crude protein; BBS, broad bean stalk; RS, rape straw; PS, paddy straw; CS, corn stalk; CHO, carbohydrates; NS, no significant.

Significant differences are indicated with capital letters in the same column and small letters in the same row; different letters mean significant difference (p<0.05).

**Table 3:** CNCPS protein fractions (DM base) of broad bean stalks, rape straw, paddy straw and corn stalk treated with *Pleurotus ostreatus* for different incubation times

Items^1)^	Substrates^2)^	Incubation times (d)					SEM	p-value

		0	7	14	21	28		
PA (%)	BBS	18.15^AB,a^	17.17^A,ab^	16.28^B,ab^	14.89^B,bc^	13.02^B,c^	0.57	<0.05
	RS	15.45^B,a^	15.15^B,a^	14.52^B,ab^	13.97^B,ab^	14.02^B,b^	0.84	NS
	PS	16.18^AB,a^	15.69^B,ab^	15.33^B,ab^	14.85^B,ab^	14.51^B,b^	0.92	NS
	CS	21.17^A,a^	20.82^A,ab^	20.16^A,abc^	19.44^A,bc^	19.71^A,c^	0.79	NS
	SEM	1.23	1.06	0.95	1.74	1.26	-	-
	p-value	<0.05	<0.05	<0.05	<0.05	<0.05	-	-
PB1 (%)	BBS	13.74^B,a^	12.89^B,ab^	12.33^B,ab^	12.45^B,ab^	11.78^B,b^	0.44	<0.05
	RS	9.92^B^	9.65^B^	9.41^B^	9.57^B^	8.89^B^	0.33	NS
	PS	10.18^B^	10.17^B^	10.12^B^	9.77^B^	9.75^B^	0.39	NS
	CS	20.15^A^	19.46^A^	19.78^A^	19.23^A^	19.95^A^	0.49	NS
	SEM	1.68	1.77	1.62	1.71	1.63	-	-
	p-value	NS	NS	NS	NS	NS	-	-
PB2 (%)	BBS	45.29^A,d^	47.57^A,cd^	49.74^A,bc^	50.40^A,b^	53.82^A,a^	0.83	<0.05
	RS	42.78^A,c^	44.02^A,bc^	44.92^A,b^	45.77^A,ab^	47.45^B,a^	0.48	<0.05
	PS	34.60^B,c^	37.18^B,bc^	37.34^B,bc^	38.62^B,ab^	40.66^C,a^	0.65	<0.05
	CS	34.97^B,c^	35.96^B,c^	37.68^B,bc^	40.06^B,ab^	41.07^C,a^	0.7	<0.05
	SEM	1.06	1.43	1.84	1.98	1.77	-	-
	p-value	<0.05	<0.05	<0.05	<0.05	<0.05	-	-
PB3 (%)	BBS	10.14^B^	9.73^B^	9.71^B^	9.93^B^	9.64^B^	0.15	NS
	RS	15.77^AB^	15.31^AB^	15.23^AB^	14.9^AB^	14.82^AB^	0.2	NS
	PS	18.49^A^	18.18^A^	18.32^A^	17.83^A^	17.56^A^	0.38	NS
	CS	16.32^AB^	15.94^AB^	15.45^AB^	14.33^AB^	14.45^AB^	0.39	NS
	SEM	1.21	1.34	1.80	1.37	1.43	-	-
	p-value	<0.05	<0.05	<0.05	<0.05	<0.05	-	-
PC (%)	BBS	12.89^B^	12.7^B^	11.96^B^	12.35^B^	11.72^B^	0.48	NS
	RS	16.1A^B^	15.86^AB^	15.95^AB^	15.8^AB^	15.83^AB^	0.44	NS
	PS	19.60^A,a^	18.98^A,a^	18.84^A,a^	18.64^A,a^	17.11^A,b^	0.58	<0.05
	CS	7.57^C^	7.84^C^	6.92^C^	6.93^C^	6.64^C^	0.31	NS
	SEM	1.71	1.74	1.83	1.67	0.63	-	-
	p-value	<0.05	<0.05	<0.05	<0.05	<0.05	-	-

CNCPS, The Cornell Net Carbohydrate and Protein System; DM, dry matter; SEM, standard error of the mean; PA, non-protein nitrogen; BBS, broad bean stalk; RS, rape straw; NS, no significant; PS, paddy straw; CS, corn stalk; PB, true protein; PB1, rapidly degradable protein; PB2, intermediately degradable protein; PB3, slowly degradable protein; PC, unavailable protein.

Significant differences are indicated with capital letters in the same column and small letters in the same row; different letters mean significant difference (p<0.05).

**Table 4:** The CNCPS carbohydrate fractions (DM base) of broad bean stalks, rape straw, paddy straw and corn stalk degraded by *Pleurotus ostreatus* for different time

Items	Substrates	Incubation times (d)					SEM	p-value

		0	7	14	21	28		
CA (%)	BBS	32.40^A,b^	32.78^A,b^	35.26^A,b^	42.18^A,a^	40.26^A,a^	1.14	<0.05
	RS	35.63^A,c^	40.36^A,ab^	40.60^A,ab^	38.80^A,b^	42.50^A,a^	0.73	<0.05
	PS	22.24^B,c^	24.58^B,bc^	28.99^B,b^	29.88^B,b^	38.53^A,a^	1.68	<0.05
	CS	30.04^A,d^	32.52^A,cd^	34.95^A,bc^	37.08^A,b^	41.77^A,a^	1.19	<0.05
	SEM	2.16	2.38	1.95	1.53	1.62	-	-
	p-value	<0.05	<0.05	<0.05	<0.05	<0.05	-	-
CB1 (%)	BBS	9.79^B,c^	10.45^B,bc^	13.50^B,ab^	13.02^B,ab^	15.71^B,a^	0.68	<0.05
	RS	8.44^B^	9.81^B^	9.18^B^	10.41^B^	9.58^B^	0.25	NS
	PS	14.72^A,b^	16.87^A,ab^	18.42^A,ab^	19.70^A,a^	20.16^A,a^	0.68	<0.05
	CS	12.16^AB^	12.61^AB^	13.87^AB^	12.13^AB^	13.44^AB^	0.36	NS
	SEM	1.68	1.53	1.47	1.98	1.72	-	-
	p-value	<0.05	<0.05	<0.05	<0.05	<0.05	-	-
CB2 (%)	BBS	14.46^B^	16.07^AB^	16.45^AB^	12.91^B^	15.79^AB^	0.46	NS
	RS	13.51^B^	14.94^B^	13.98^B^	13.89^B^	15.88^AB^	0.44	NS
	PS	13.57^B,a^	10.45^B,b^	7.63^C,b^	8.16^BC,b^	10.11^B,b^	0.66	<0.05
	CS	23.79^A^	23.41^A^	24.17^A^	24.87^A^	21.40^A^	0.46	NS
	SEM	1.16	1.68	1.36	1.29	1.34	-	-
	p-value	<0.05	<0.05	<0.05	<0.05	<0.05	-	-
CC (%)	BBS	43.37^A,a^	40.69^B,a^	34.78^B,b^	31.89^B,bc^	28.23^B,c^	1.57	<0.05
	RS	42.40^A,a^	34.90^BC,bc^	35.89^B,bc^	36.93^AB,b^	32.02^A,c^	1.01	<0.05
	PS	49.46^A,a^	48.09^A,ab^	44.95^A,bc^	42.24^A,c^	31.22^A,d^	1.79	<0.05
	CS	34.00^B,a^	31.45^C,a^	27.04^C,b^	25.93^BC,bc^	23.38^B,c^	1.1	<0.05
	SEM	2.34	1.59	1.68	2.15	1.43	-	-
	p-value	<0.05	<0.05	<0.05	<0.05	<0.05	-	-

CNCPS, The Cornell Net Carbohydrate and Protein System; DM, dry matter; SEM, standard error of the mean; CA, fast degradable carbohydrate; BBS, broad bean stalk; RS, rape straw; PS, paddy straw; CS, corn stalk; NS, no significant; CB1, intermediate degradable carbohydrate; CB2, slowly degradable carbohydrate; CC, unavailable degradable carbohydrate.

Significant differences are indicated with capital letters in the same column and small letters in the same row; different letters mean significant difference (p<0.05).
